# Assessment of Transoral Robotic Surgery (TORS) Content on YouTube

**DOI:** 10.7759/cureus.63857

**Published:** 2024-07-04

**Authors:** Mohammed D Akbar, Abdul K Taufique, Daniyaal A Kamran, Abdul R Harris, James Lyons

**Affiliations:** 1 College of Medicine, Alabama College of Osteopathic Medicine, Dothan, USA

**Keywords:** transoral robotic surgery, sleep apnea surgery, adult education, youtube study, otolaryngology education

## Abstract

Background: Online video hosting websites such as YouTube have been increasingly used by medical institutions to spread information about new and exciting topics. However, due to the large number of videos uploaded daily and the lack of peer review, few attempts have been made to assess the quantity and quality of information that is uploaded on YouTube. For this study, our team assessed the available content on the transoral robotic surgery (TORS) procedure.

Methods: A qualitative case study model was employed. Videos related to TORS were collected using a unified search protocol. Each video was then analyzed, and metrics of the following data points were collected: views, likes, comments, upload date, length of video, author type, author, and region of origin. Each dataset was analyzed by two distinct authors, and interrater reliability was calculated. Quantitative and qualitative statistics were curated.

Results: A total of 124 videos were analyzed for this review. The breakdown of videos was as follows: 15.32% (19) in the educational for patients category, 16.94% (21) in the educational for trainees category, 30.65% (38) in the procedural overview category, 8.87% (11) in the patient experience (PE) category, 10.48% (13) in the promotional category, 12.10% (15) in the other category, and 5.65% (7) in the irrelevant (IR) category. The total number of views across all videos analyzed was 2,589,561. The total number of likes was 14,827, and the total number of comments was 2,606. The average video length was 8.63 minutes. The most viewed category was the PE category at 1,014,738 and the most liked at 1,714. The least viewed category was IR at 21,082. The PE category had the most engagement based on combined comments and likes. The most watched video, with 774,916 views, was in the PE category under the “TORS for Thyroidectomy” search term and was titled “Thyroid Surgery (Thyroidectomy).”

Conclusion: As the prevalence of online videos regarding medical devices, procedures, and treatments increases, patients and trainees alike will look toward resources such as YouTube to augment their understanding. Patients, providers, and medical education platforms should take heed of the promise and pitfalls of medical content on YouTube.

## Introduction

Transoral robotic surgery (TORS) has shown promise in the treatment of a variety of medical conditions, including head and neck cancer, sleep apnea, and more. It offers greater visibility, ease, and a shorter length of procedure than standard therapies [1]. As with any new technique or topic, both patients and providers alike are increasingly turning to the Internet for more information, highlighting the need for high-quality, peer-reviewed content [2-4].

One common location for video-based content is YouTube. YouTube is the largest open-access video platform on the Internet, boasting over 2.6 billion users. It is home to a broad swath of different types of content, from entertainment to education. In particular, academia has realized the utility of such a website in spreading, sharing, and building upon knowledge and research [5]. Previous research has been conducted on the application of YouTube videos as a learning tool for hip arthroscopy, neurosurgery, brachytherapy, facelifts, ear tube surgery, H1N1 flu, and more [6-11]. One noted weakness of YouTube as a platform for educational content is the absence of a robust system of authentication and verification of published material. There have been documented cases of misinformation on health-related topics in the past [12].

To date, no study has assessed the content available on YouTube regarding TORS. The present study aims to assess the quality and quantity of available content on YouTube regarding TORS and characterize how accessible the available content is to those who are not healthcare professionals, such as patients looking to gain more information via YouTube. Finally, the present study aims to highlight gaps in available content on YouTube, hoping that academic media publishers can focus on these areas in the future.

## Materials and methods

Regarding TORS videos on YouTube, four categories of interest were assessed (TORS head and neck cancer, tonsillectomy, sleep apnea, and thyroidectomy) using the following four search terms: “Trans oral robotic surgery for sleep apnea,” “Trans oral robotic surgery for tonsillectomy,” “Trans oral robotic surgery for head and neck cancer,” and “Transoral robotic surgery for thyroidectomy.” All data collected for this study were from publicly available videos on the YouTube website (www.youtube.com), and as such, this study was exempt from institutional review board approval. Each author conducted a respective video search independently for one of the search terms above, sorted the search list using the “Sort by Relevance” filter, and classified each video. A second, different author also classified each video, and data regarding agreement were used to calculate interrater reliability (Table 1). Disputes with classification between authors were resolved through discussion, and if consensus could not be reached, a third author classified the video.

**Table 1:** Assessment of interrater reliability among authors

Rater 1	Rater 2	Kappa coefficient	Standard error
MA	AK	0.65	0.09
MA	AH	0.71	0.09
MA	DK	0.66	0.9
AK	AH	0.75	0.08
AK	DK	0.73	0.09
DK	AH	0.58	0.1

The videos found under each search term were grouped based on the category that each video was presented as follows: “educational for patients (EDP),” “educational for trainees (EDT),” “procedural overview (PO),” “patient experience (PE),” “promotional (PRO),” “other (O),” and “irrelevant (IR)” to that particular search term. Videos that contained material that plausibly belonged to multiple categories were placed in the category that characterized the majority of the video’s content as determined by an author. Inclusion criteria were not used, as we opted to create a separate category, “IR,” for videos unrelated to the search term. Exclusion criteria included videos not in English, duplicate videos, and videos without audio. Search results from the first three pages of the YouTube searches for each search term were included for analysis, as previous research has shown that the majority of users only search through the first three pages of results [13].

Variables that were extracted from each video include the following: number of views, number of likes, number of comments, length of video (LOV; in minutes), upload date, author of the video, and region of origin. Descriptive statistics were performed with Microsoft Excel software (Microsoft Corporation, Redmond, WA).

## Results

Section focus: search results by year

**Figure 1:**
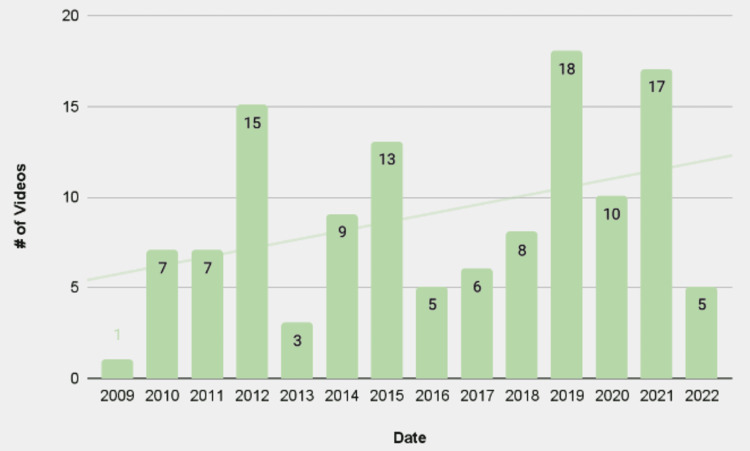
Search results by year An upward trend in the number of search results is observed during the period from 2009 to 2022

Upload trends related to the relevant TORS search terms were assessed for video uploads between 2009 and 2022, highlighting the increased utilization of the social media platform YouTube by content creators to present information about TORS (Figure 1).

Section focus: category data

**Figure 2:**
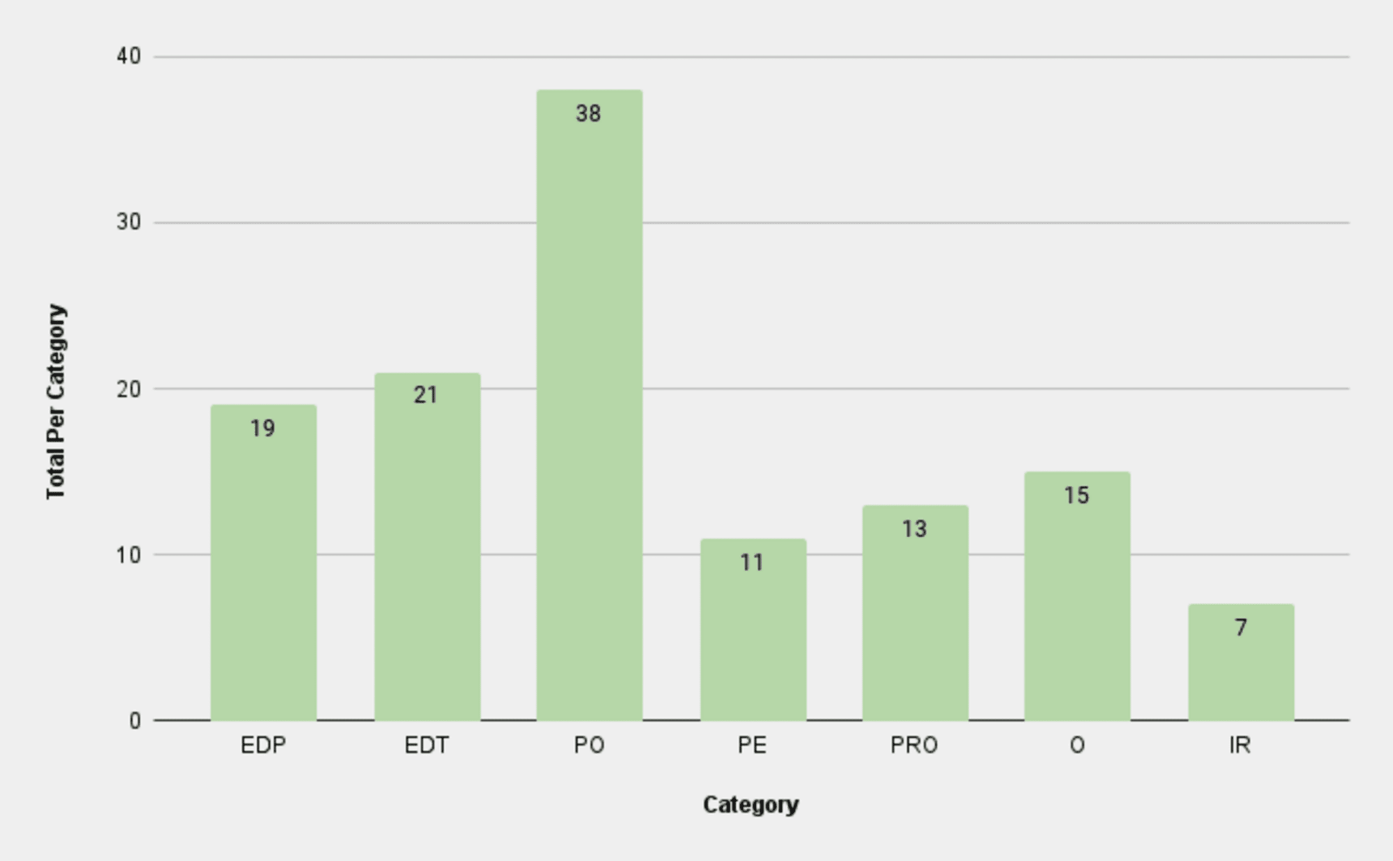
Total videos per category across all search terms EDP: educational for patients; EDT: educational for trainees; PO: procedural overview; PE: patient experience; PRO: promotional; O: other; IR: irrelevant

The categories analyzed were the following: EDP, EDT, PO, PE, PRO, O, and IR. Overall, PO was the most populated category across the various search terms (Figure 2), although for the search term “TORS for Thyroidectomy,” PO was second to the O category. The proclivity toward highly specialized content on surgical procedural techniques suggests that these videos were geared toward medical professionals rather than patients. In the same vein, PE was the second lowest overall when looking at the aggregate data of all the search terms. The second highest scoring category was EDT, which is also geared toward medical professionals. Patient-oriented information concerning the TORS procedures was found less often. In addition, less material was put out by medical establishments tackling patient education.

Section focus: LOV

**Figure 3:**
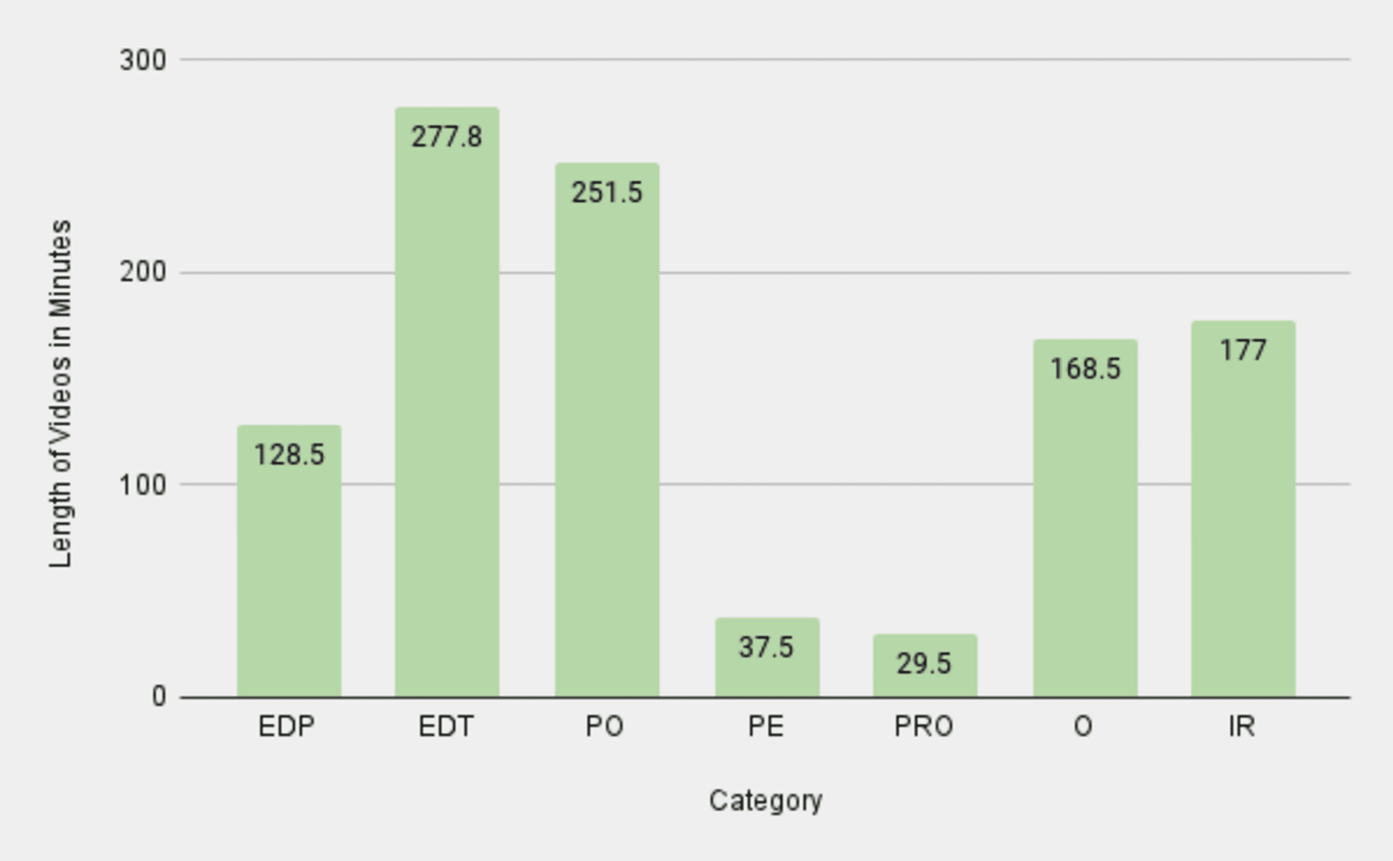
LOV totals by category EDP: educational for patients; EDT: educational for trainees; PO: procedural overview; PE: patient experience; PRO: promotional; O: other; IR: irrelevant; LOV: length of video

**Figure 4:**
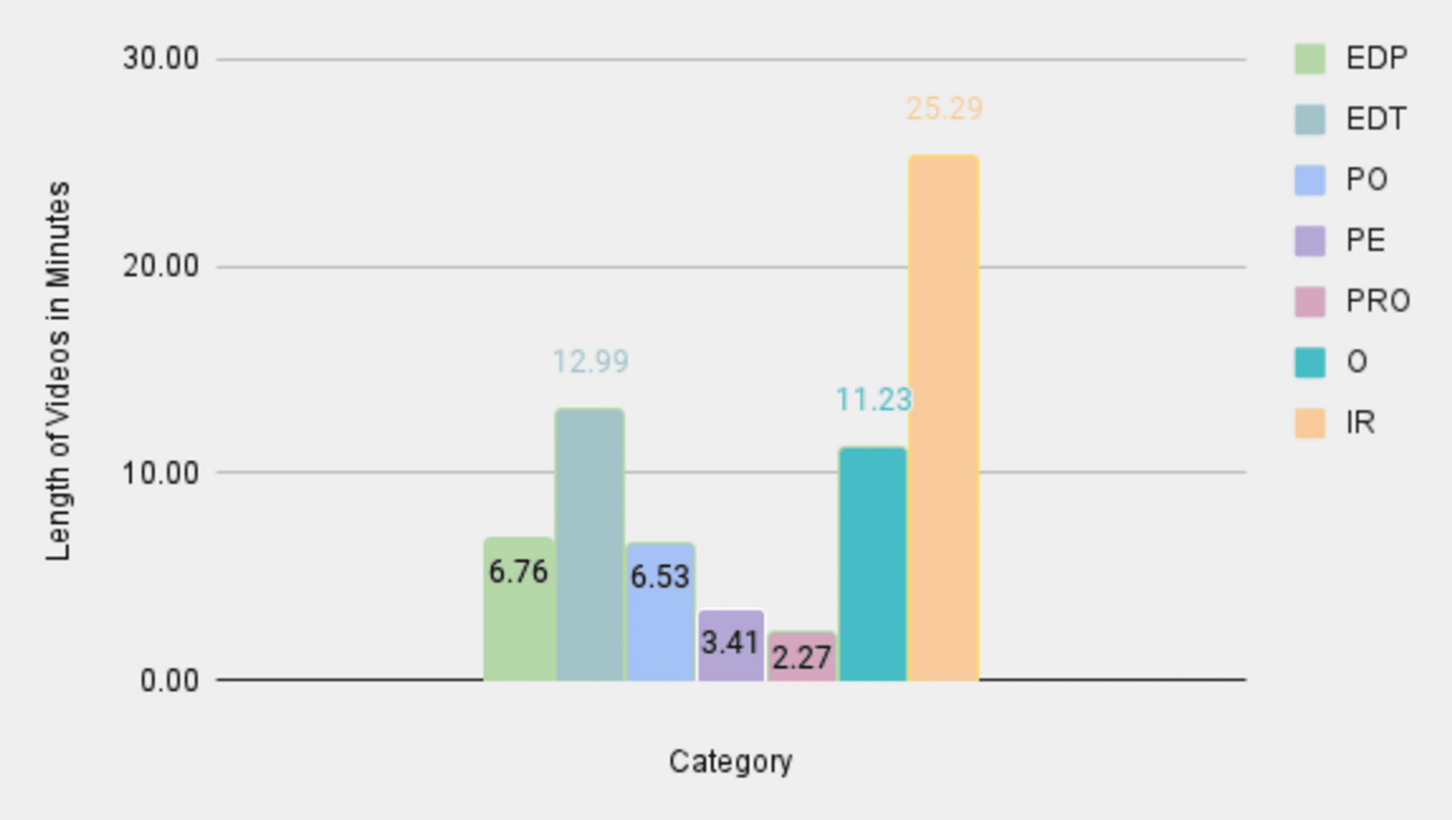
Mean LOVs EDP: educational for patients; EDT: educational for trainees; PO: procedural overview; PE: patient experience; PRO: promotional; O: other; IR: irrelevant; LOV: length of video

**Figure 5:**
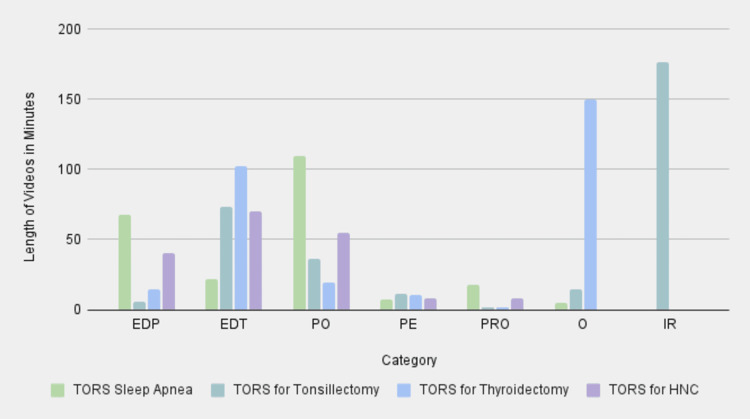
Total video lengths categorized to each TORS subset EDP: educational for patients; EDT: educational for trainees; PO: procedural overview; PE: patient experience; PRO: promotional; O: other; IR: irrelevant

To analyze the LOV data for different TORS procedures, the authors collected data points for each category that were relevant to each surgery. The overall screen time of the videos was the longest for the EDT category, with PO following shortly after (Figure 3). This suggests that a majority of the videos on these specific surgeries are intended for an already educated individual on this subject matter, not necessarily targeting a patient demographic. While such videos are helpful for individuals who practice TORS or are completing training in TORS, it seemed that PE videos lacked in length and depth of knowledge, likely failing to properly prepare patients to make an informed decision regarding TORS. The mean LOVs across all search terms were highest for the IR category, followed by the EDT and O categories, which were substantially less lengthy on average (Figure 4). When the total video lengths were subcategorized by surgical indication, some patterns emerged (Figure 5). The majority of the minutes for thyroidectomy and tonsillectomy search terms were classified as O and IR, respectively, thus revealing a dearth of content in this area for patients. The sleep apnea search term had the highest screen time in the PO category, highlighting a specific interest in using TORS for this particular indication. Finally, the relative lack of PE videos is consistent across all search terms, which corroborates with previous results.

Section focus: comments by category

**Figure 6:**
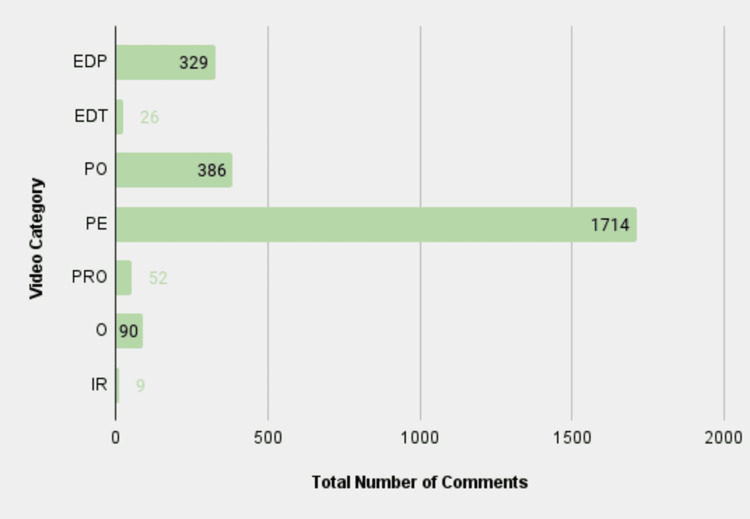
Total number of comments for each video category EDP: educational for patients; EDT: educational for trainees; PO: procedural overview; PE: patient experience; PRO: promotional; O: other; IR: irrelevant

Another prominent aspect of our analysis concerns one of the primary methods of engagement on the YouTube platform: comments. They serve as an important metric to gauge the level of engagement that the videos receive. The authors assessed the number of comments across all the different categories. It was found that the PE category had the most comments under its videos by far, at 1,714 comments. The PO category had the second most comments with 386, followed by EDP with 329. Interestingly, the O category, PRO category, EDT category, and IR category all had less than 100 comments each (Figure 6). The large gap between comments on TORS videos related to PE and other search terms underlines the disproportionate extent of patient engagement on patient-centered videos, which belies its relatively low average and total video length.

**Figure 7:**
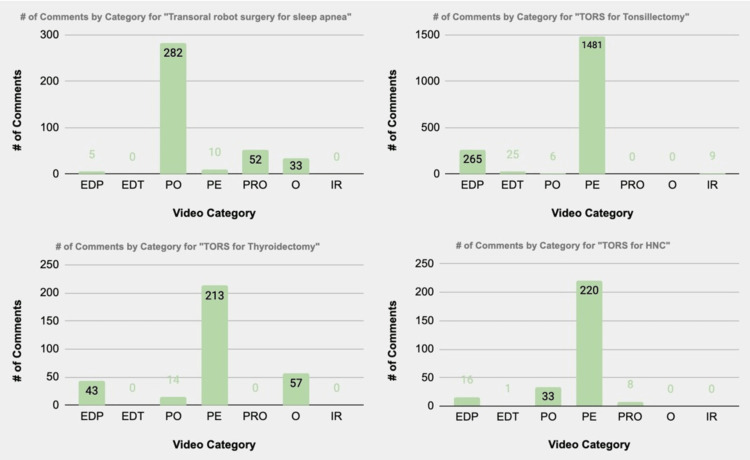
Number of comments by category, separated by search term EDP: educational for patients; EDT: educational for trainees; PO: procedural overview; PE: patient experience; PRO: promotional; O: other; IR: irrelevant

For the “TORS for sleep apnea” search term, the PO video category had the most comments at 282. However, for the rest of the search terms, the PE video category had the most comments (Figure 7). Perhaps, due to the novelty of using TORS for sleep apnea indication, more medical professionals are interested in an overview of how the TORS procedure is performed for sleep apnea, compared to the number of people interested in the patient’s experiences with this procedure. For the other TORS search terms, viewers were more willing to comment on PE videos, which are broader in scope than POs. This finding could suggest that viewers interested in the TORS PE are more likely to comment and engage with these videos than those intended for medical professionals.

Furthermore, regarding comments by category, it is observed that commenters utilized videos that were intended for patients the most. Comments on perceived outcomes of the TORS surgeries tended to be positive overall for most of the TORS indications. Nevertheless, studies indicate that individuals on social media strive to present a positive self-image online [14]. This observation could be explored in future studies, as PE data can be collected from YouTube and other platforms to gain beneficial information for other patients and surgeons.

One pertinent observation is that videos that were highly academic, more specialized, and for medical professionals had fewer comments. This information suggests that the academic and medical professional communities do not prioritize engagement on YouTube videos and could signal an area of improvement for medical education videos published by institutions. More work must be done to explore this relationship, as YouTube and online videos could become potent educational tools.

## Discussion

The present study aimed to classify the available content on the online video-sharing platform YouTube regarding the TORS procedure. Our analysis of the category data showed that a majority of the videos available on TORS were geared toward medical professionals rather than patients. Less patient education material was published by medical establishments, and less information geared toward patients regarding TORS was available. One possible explanation is that medical professionals and institutions are utilizing online video platforms primarily for medical education purposes and underutilizing them when it comes to patient education and experience. Across the board, there has been a push for greater involvement of such institutions in producing patient-oriented medical content, even at the level of state health departments [15].

Looking at the LOVs available on TORS yielded additional insights. It was found that many of the videos had increased length of time when geared toward education for a trainee/physician, suggesting that a majority of the videos on TORS on YouTube are intended for healthcare professionals who are well-versed in the subject matter and not necessarily for patient education. While such videos are certainly helpful for healthcare professionals, PE videos lack the necessary length and depth of knowledge that would help patients to properly make an informed decision on the treatment or other alternatives. This serves to highlight an area of improvement for medical institutions that offer TORS moving forward.

Furthermore, looking at the number of comments per video revealed trends like viewer engagement with TORS-related video content on YouTube. TORS videos related to PE generally had more comments than videos that were for medical professionals. Differences in commenting patterns between specific TORS search terms were also present. Perhaps due to the relative novelty of the TORS procedure for sleep apnea, there was more viewer engagement in PO videos than videos on patients’ experiences with this procedure. For the other TORS search terms, viewers were more willing to comment on PE videos, which are broader in scope than POs. This is likely owing to the established nature of using TORS for these indications. This finding suggests that viewers who are interested in the TORS PE are more likely to comment and engage with these videos than viewers of videos that are intended for medical professionals.

Finally, after looking at the comments by category, we can see that commenters utilized the videos that were intended for patients the most. Videos that were published by academic institutions for medical education and training received fewer comments. This information suggests that academic and medical professional communities do not prioritize engagement on YouTube videos and could signal an area of improvement for medical education videos published by institutions.

One weakness of the current study is the inherent subjectivity of the categorization method, which affects interrater reliability. To combat this, the authors sought to establish objective markers for categorizing videos that were consistent across each category. Additionally, in the present study, we focused strictly on videos that were available on YouTube. Similar studies conducted on other video streaming platforms were not available. One assumption that was made before undertaking our analysis is that the videos are being viewed by their intended audience, which may not be necessarily true. Search engine optimization algorithms that platforms like YouTube employ can skew engagement, views, and other metrics.

## Conclusions

In the present study on TORS, the category with the highest engagement statistics (views, likes, and comments) was PE. This is even though only 8.87% of videos assessed fell in this category, which highlights the significant need for patient-oriented content on YouTube regarding TORS.

There is also a discrepancy in engagement between videos from academic and medical institutions geared toward medical professionals and videos regarding PEs. According to the present study, there is a quantifiable lack of material regarding PE with TORS on YouTube. Additionally, there is a mismatch in the engagement of the medical community with certain videos that are popular with the layperson community. This serves as an area of focus in the future development of patient-oriented medical content on YouTube.
